# Working memory deficits in adults with ADHD: is there evidence for subtype differences?

**DOI:** 10.1186/1744-9081-2-43

**Published:** 2006-12-15

**Authors:** Julie B Schweitzer, Russell B Hanford, Deborah R Medoff

**Affiliations:** 1 Division of Child Psychiatry, Department of Psychiatry, 737 W. Lombard St., Suite 442, University of Maryland School of Medicine, Baltimore, Maryland, 21201, USA; 2 Department of Psychology, 532 Kilgo Circle, Emory University, Atlanta, Georgia, 30322, USA; 3 Division of Services Research, Department of Psychiatry, 737 W. Lombard St., University of Maryland School of Medicine, Baltimore, Maryland, 21201, USA

## Abstract

**Background:**

Working memory performance is important for maintaining functioning in cognitive, academic and social activities. Previous research suggests there are prevalent working memory deficits in children with attention deficit hyperactivity disorder (ADHD). There is now a growing body of literature characterizing working memory functioning according to ADHD subtypes in children. The expression of working memory deficits in adults with ADHD and how they vary according to subtype, however, remains to be more fully documented.

**Methods:**

This study assessed differences in working memory functioning between Normal Control (NC) adults (N = 18); patients with ADHD, Combined (ADHD-CT) Type ADHD (N = 17); and ADHD, Inattentive (ADHD-IA) Type (N = 16) using subtests from the Wechsler Adult Intelligence Scale-III and Wechsler Memory Scale-III and the Paced Auditory Serial Addition Task (PASAT).

**Results:**

The ADHD groups displayed significant weaknesses in contrast to the NC group on working memory tests requiring rapid processing and active stimulus manipulation. This included the Letter-Number-Sequencing test of the Wechsler scales, PASAT omission errors and the longest sequence of consecutive correct answers on the PASAT. No overall ADHD group subtype differences emerged; however differences between the ADHD groups and the NC group varied depending on the measure and the gender of the participants. Gender differences in performance were evident on some measures of working memory, regardless of group, with males performing better than females.

**Conclusion:**

In general, the data support a dimensional interpretation of working memory deficits experienced by the ADHD-CT and ADHD-IA subtypes, rather than an absolute difference between subtypes. Future studies should test the effects of processing speed and load on subtype performance and how those variables interact with gender in adults with ADHD.

## Background

Attention deficit hyperactivity disorder (ADHD) may be the most common childhood DSM-IV diagnosis [1], with 30% to 50% of cases persisting into adulthood [2]. Research in pediatric ADHD has progressed to documenting differences in neuropsychological functioning at the level of the DSM-IV subtypes: *Combined *(ADHD-CT, both inattentive and hyperactive-impulsive symptoms), *Predominantly Inattentive *(ADHD-IA), and least common, *Predominantly Hyperactive-Impulsive *(ADHD-HI). The psychiatric classification system for ADHD, with its various subtypes, has evolved substantially over the years. DSM-IV defined the disorder based on the best available research at the time, but there is debate as to whether the exiting subtypes provide an accurate description of the disorder. Some researchers [2-4] hypothesize that the ADHD-IA subtype may constitute a different type of attention disorder compared to the ADHD-HI and ADHD-CT subtypes. Barkley [2] is most associated with this view. He conceptualizes the ADHD-HI and ADHD-CT as, centrally, exhibiting deficits in behavioral inhibition that in turn lead to impairment in executive functioning and working memory (WM). According to this hypothesis the ADHD-IA subtype is a fundamentally distinct disorder with problems in attention arising from noninhibitory mechanisms. In contrast to this hypothesis, others note (e.g., [5] that the ADHD-CT and ADHD-IA subtypes both share inattention as a common dimension and therefore the ADHD-CT and ADHD-IA should both be impaired on tests requiring attention, processing speed, vigilance and WM. Indeed, there is evidence that inattentive symptoms are most associated with neuropsychological impairment across the ADHD-CT and ADHD-IA subtypes [5]. Thus, one could conclude that individuals with the ADHD-CT may be most impaired because they experience the combined symptoms of inattention plus inhibition.

The results for documenting neuropsychological differences according to DSM-IV nosology for ADHD subtypes have been ambiguous at best. Several pediatric studies found no or limited differences between ADHD-CT and ADHD-IA subtypes on a series of executive functioning measures [3,6-8]. However, other researchers have detected subtype differences or demonstrated differential performance across subtypes when making comparisons to control groups [9,10].

While the findings regarding subtype differences in children are mixed, even less is known about the presence of neuropsychological differences between subtypes among ADHD adults. Gansler et al. [11] compared ADHD-HI to ADHD-IA adults and found that the types of executive functioning deficits exhibited differed across groups. The ADHD-HI group showed perseverative responding on the Wisconsin Card Sort Test, while the ADHD-IA group demonstrated difficulty in WM on the Auditory Consonant Trigrams (ACT) Test. Also, the ADHD-IA group emitted a higher rate of Continuous Performance Test (CPT) commission errors, a somewhat counterintuitive finding because one would tend to associate such errors with impulsivity rather than inattention.

Murphy and colleagues [12] examined neuropsychological functioning in a large sample of ADHD adults. The authors found no subtype differences on tests measuring interference control, inattention, response inhibition, WM, and verbal-ideational fluency. They suggest that the disparity between their results and Gansler's [11] may reflect 1) differences in the WM and response inhibition tasks used; or 2) problems related to the current DSM-IV diagnostic criteria in which adults who formerly may have been classified as ADHD-CT as children, are diagnosed with the ADHD-IA subtype as adults due to a reduction of the hyperactive/impulsive symptoms and thereby no longer meet criteria for the ADHD-CT subtype.

The inclusion of WM subtests in the battery of neuropsychological tests in previous studies in ADHD is quite logical given the growing body of literature suggesting WM impairments associated with the disorder [2,13-18]. WM, or the ability to hold information in mind, manipulate it, and use it to guide behavior, is a key component of executive functioning. A recent meta-analysis of neuropsychological performance in adults with ADHD [19] found effect sizes for tests measuring WM and verbal memory to be among the highest within 10 functional domains. Gallagher and Blader [20] suggest that "stressful" WM tests may be more sensitive to detecting deficits in adults with ADHD. Specifically, they highlight studies using the Paced Auditory Serial Addition Task (PASAT) [21,22]. This test requires active manipulation of stimuli in which an action is required on the presented stimuli. This is in contrast to short-term memory tasks where previously presented stimuli are simply reiterated. The PASAT already has been shown to discriminate between a sample of male ADHD-CT adults and controls on a behavioral and functional neuroanatomical level [23,24]. A review of neuropsychological performance in adults with ADHD [17] found the PASAT produced one of the largest effect sizes in comparisons between controls and ADHD adults. Yet, we do not know if the PASAT is useful for discriminating between subtypes. One reason the PASAT may be more sensitive to detecting subtype differences is its ability parametrically to assess processing speed capabilities. It does this by using varying interstimulus intervals (ISI) between stimulus presentations.

We expect that both ADHD subtypes will exhibit impaired processing speed given its relationship to inattention [5], but that the ADHD-IA group will be more impaired by shorter ISIs reflective of greater processing speed difficulties given previous research [5,25]. Similarly we expect both subtypes to produce the most common type of error on the PASAT [26], failures to respond, or omission errors due to inattention. The number of consecutive correct answers, or Longest Sequence of Consecutive Correct Answers, is another measure of WM functioning on the PASAT that appears to be a sensitive measure of functioning in both ADHD [27] and neurologically-impaired populations [28-30]. We hypothesize that the ADHD-CT type will perform more poorly on this measure than the ADHD-IA type because this measure will be sensitive to errors reflective of response inhibition and inattention.

This study also tested for gender differences in WM performance. The ADHD literature tends not to find significant differences between adult males and females [31-33] or boys and girls with ADHD [34], but only a few studies [8,35,36] have examined the effect of gender on executive functioning performance. There is, however, some suggestion of gender differences according to subtype on symptom comorbidity [37], neuropsychological functioning [25], and motor performance [38].

Gender differences in WM paradigms are relatively sparse with most studies examining memory differences in relationship to gender, rather than explicitly studying WM performance. Recent brain imaging studies, however, have begun to explore gender differences in relationship to brain activation [39-41] and found that gender can significantly affect brain activation. An fMRI study [41] suggests that men and women may use different neural substrates and perhaps strategies to perform WM tasks. Speck et al. [41] found that women performed more accurately but also more slowly in comparison to the male participants on WM paradigms. These data suggest that WM tasks with a speed component may be more influenced by gender. Furthermore, there were strong lateralization differences with male participants displaying symmetric activation or right brain dominance, versus female participants who were more likely to activate the left hemisphere during the WM tasks. The authors concluded that the differences in lateralization are due to either differences in the use of problem-solving strategies by gender or differences in the neuroanatomy used to solve the tasks. We decided to explore gender differences in this study given that there is also some evidence for lateralization differences in how individuals with ADHD perform WM tasks [24] and thus, it is plausible that ADHD may interact with gender on executive functioning performance.

The objectives of this study are to test for differences in WM between adults with and without ADHD and between ADHD-CT and ADHD-IA adults. We hypothesize that the ADHD participants will exhibit WM deficits in contrast to healthy control (NC) participants, and that there will be subtype-specific WM errors. We hypothesize that gender will have a significant effect on WM tasks, particularly those that require a greater processing speed load. It is also expected that faster interstimulus interval (ISI) presentation rates on the PASAT will be more sensitive in detecting differences between the NC and ADHD groups due to demands in processing speed.

## Methods

### Participants

The sample included a total of 51 participants: 17 Combined Types (ADHD-CT, 11 male), 16 Inattentive Types (ADHD-IA, 10 male); and 18 Normal healthy Controls (NC, 13 male). See Table 1 for group averages of age and IQ. The three groups did not differ significantly across age, education or IQ, nor did the subjects differ in these areas by gender (Table 2) or in gender by group analyses.

**Table 1:** Mean age, educational level and full scale IQ by group

	ADHD-CT (*n *= 17)		ADHD-IA (*n *= 16)		NC (*n *= 18)			
					
Measure	M	SD	M	SD	M	SD	*F*(2,48)*	Sig.
Age	33.35	11.45	36.44	10.73	31.94	7.70	0.88	0.42
Education (years)	16.32	3.40	15.28	2.18	16.97	2.19	1.74	0.19
Full scale IQ	114.18	8.29	116.31	14.34	121.89	11.70	2.05	0.14

*p < 0.05 for *F *statistic.

**Table 2:** Mean age, educational level and full scale IQ by gender

	Males (*n *= 34)		Females (*n *= 17)			
				
Measure	M	SD	M	SD	*t*(49)*	Sig.(2-tailed)
Age	34.18	9.43	33.12	11.35	0.35	0.72
Education (years)	16.32	2.79	16.03	2.57	0.35	0.72
Full scale IQ	117.47	11.29	117.76	13.38	0.08	0.93

*p < 0.05 for *t *statistic.

### Recruitment and screening measures

Participants were recruited from a university-based adult ADHD clinic and local advertisements. Following a description of the study and its associated risks, each participant gave written informed consent for a protocol approved by the Human Investigations Committee associated with the university at which the study was conducted.

Each potential participant completed a personal information questionnaire packet (PIQ) that inquired about the volunteer's developmental, health, medication, alcohol use, employment, social, financial, and educational history. Potential participants also completed a computerized structured psychiatric interview (Mini-SCID for DSM-IV)[42] and the Symptom Checklist-90, Revised (SCL-90-R)[43] to screen for psychiatric conditions. The Wechsler Adult Intelligence Scale – 3^rd ^Edition (WAIS-III)[44] screened for intellectual impairment.

Volunteers completed two self-report versions of the Adult ADHD DSM-IV Rating Scale [45], one reviewing current behavior and the second considering behavior between the ages of 5 and 12. Volunteers also had another adult (e.g., spouse, close friend) rate their current behavior. Similarly, a retrospective fourth and (when available) fifth scale were completed by someone older than the volunteer who knew him or her as a child. Preference was to obtain these ratings from a mother and father when available. In five instances, a grandmother or older brother completed the ratings when fathers were unavailable. When indicated, follow-up interviews with parents were conducted to clarify prior history, course of symptoms, and to distinguish between subthreshold ADHD-CT versus participants with ADHD-IA. Screening for the presence or absence of ADHD also included a semi-structured interview based on the DSM-IV criteria for ADHD [46] and ratings by one of the investigators on the Adult ADHD DSM-IV Rating Scale [45].

### Exclusion criteria

Prospective participants were excluded for the following reasons: clinically significant medical conditions, mental retardation, clinically unstable psychiatric conditions (psychosis, criminality, suicidal behaviors), bipolar disorder, obsessive-compulsive disorder, current major depressive episode, drug or alcohol abuse or dependence within one year preceding the study, and/or current use of antipsychotic medication. In addition, healthy normal controls (NC) were excluded if they met DSM-IV criteria for ADHD, learning disability or any major psychiatric disorder. No participant was taking psychoactive medication that would impact attention at the time of testing. Participants taking stimulants took a "medication vacation" for at least 24 hours before testing.

### Diagnostic procedure

The investigators first screened prospective participants by phone to obtain a general understanding of the severity and chronicity of the volunteer's ADHD symptoms and presence of exclusionary criteria. Qualifying prospective participants then completed a research packet with screening measures to bring to the first visit. Next, a master's level clinician (R.B.H.) and/or a licensed, Ph.D.-level psychologist (J.B.S.) interviewed each volunteer. J.B.S. participated in 70% of the interviews of those accepted into the study and reviewed all interview forms and test records. Interviews included a review of the PIQ, the semi-structured interview based on the DSM-IV criteria for ADHD [46], and items endorsed on the mini-SCID and SCL-90-R. The interview also included a review of any possible discrepancies between the Adult ADHD DSM-IV Self-Current, Self-Retrospective, Other-Current, and Other-Retrospective Rating Scales with the participant. The investigators contacted informants who provided discrepant ratings to assist in determining the validity of the ratings.

Procedurally, the first step toward a diagnosis for either subtype of ADHD required significant symptom reports on the ADHD self-rating scale with ratings of a 2 (frequent) or 3 (very frequent) on the appropriate scale(s) for either subtype for 6 or greater symptoms (see Table 3 for ADHD symptom ratings by scale for each group). The next step required a review of ratings from spouses/friends and parents/grandparents/older siblings. Investigators only retained volunteers with third party rating scales corroborating impairment. Third, results from the semi-structured DSM-IV [46] interviews reviewing the ADHD criteria for the Predominantly Inattentive Type (ADHD-IA) and Combined Type (ADHD-CT) informed the process for each potential participant. Finally, the investigators combined the rating scale and ADHD DSM-IV interview results with information derived from the PIQ, mini-SCID and SCL-90-R to determine whether current impairment from the symptoms was present in two or more settings, had a clinically significant impact on social, occupational, and/or vocational functioning, and could not better be accounted for by another mental disorder. Participants who met all the above criteria continued in the study. Similarly, all control volunteers were required to complete the same diagnostic procedure to rule out the presence of ADHD or other psychiatric or learning disorders.

**Table 3:** 

Subject Type	Males					Females				
	Inattentive Symptoms			Hyperactive-Impulsive Symptoms		Inattentive Symptoms			Hyperactive-Impulsive Symptoms	

	*N*	Mean	*SD*	Mean	*SD*	*N*	Mean	*SD*	Mean	*SD*
		
NC	13	0.00	0.00	0.38	0.65	5	0.00	0.00	0.20	0.45
ADHD-IA	10	7.30	1.34	2.10	1.45	6	8.00	1.26	1.83	1.17
ADHD-CT	11	7.27	1.79	6.91	2.07	6	7.50	1.52	6.67	1.21

*Adult ADHD DSM-IV Rating Scale [40]

*Note*. NC = normal control, ADHD-IA = inattentive type, ADHD-CT = combined type.

### WM measures

Participants completed Digit Span (DS), Arithmetic, and Letter-Number-Sequencing (LNS) subtests from the WAIS-III and the Spatial Span subtest of the Wechsler Memory Scale – 3^rd ^Edition (WMS-III). The PASAT [47] further assessed WM via computer, aurally presenting single digit numbers every few seconds. The participant was instructed to add each number to the preceding number (i.e., the second to the first, the third to the second) and to vocalize the sums. Four trials of 50 digits each were given at four presentation rates: 2.4, 2.0, 1.6, and 1.2 s per digit. This report presents the total Number Correct responses summed over the trials to assist in comparing to the majority of other studies using the PASAT [26]. The Longest Sequence of Consecutive Correct Answers measures PASAT performance that is more sensitive to "chunking strategies" than the total number of correct responses. Chunking can occur when a participant attempts to lessen the WM load by adding consecutive numbers and then skipping some number pairs in order to "catch up" until the next string of numbers can be added together. The Longest Sequence measure theoretically enables one to measure how long the participant was able to maintain set, resist distractors and stay apace with the speed requirements. This measure [27] has shown to be a useful measure of PASAT performance in previous ADHD studies. Omission errors are included as a measure of lapses in attention where no responses are made. We measured the effects of varying interstimulus interval (ISI) presentation rate on performance to assess for differences in processing speed ability between groups.

### Analyses

An analysis of variance (ANOVA) tested for group differences between gender, NCs and the two ADHD groups (ADHD-CT & ADHD-IA subtypes) on the age-corrected LNS, Spatial Span, Arithmetic, and DS followed by least significant difference (LSD) post hoc pairwise comparisons for significant omnibus group effects. Covariates included age and education for the Wechsler subtests. A mixed design with between-group (gender, group) and within-subject repeated (ISI presentation rates) factors tested for performance differences on the PASAT for each of the three dependent measures labeled Number Correct, Omission Errors, and Longest Correct Sequence. IQ subtests (Block Design and Vocabulary) unlikely to be influenced by attention also were specified as covariates of interest for the PASAT because performance on the PASAT may be associated with age and intelligence [48,49]. Post hoc pairwise comparisons were also used to inspect any significant main effect differences on the PASAT variables. A Greenhouse-Geisser corrected univariate repeated measures ANOVA tested for significant effects associated with presentation rate (ISI) on performance. In all analyses, the level of significance was set at *p *= .05. Effect sizes are presented for the comparisons using partial eta squared (η_p_^2^) as the statistic. Partial eta-squared is the proportion of variance accounted for attributable to a given effect, partialling out all other factors in the model [50].

## Results

### Evaluation of gender differences

Across all groups (ADHD-CT, ADHD-IA, and NC), males performed significantly better than females on some WM measures. There was a significant main effect for Gender, (*F*(1,43) = 5.33, *p *= 0.03, η_p_^2 ^= 0.11) on the LNS. Men (*M *= 13.21, *SE *= 0.56) performed more trials correctly than women (*M *= 11.06, *SE *= 0.55). Differences between men and women approached significance on Digit Span (*F*(1,43) = 3.67, *p *= 0.06, η_p_^2 ^= 0.08), once again with men (*M *= 12.62, *SE *= 0.48) performing better than women (*M *= 11.12, *SE *= 0.64). On the PASAT, significant main effects for Gender emerged on Number Correct, (*F*(1, 38) = 5.96, *p *= 0.02, η_p_^2 ^= 0.13), on which men (*M *= 36.42, *SE *= 1.57) answered significantly more items correctly than women (*M *= 30.02, *SE *= 2.2). Significant main effects for Gender also emerged on the PASAT Omission Error variable (*F*(1, 38) = 4.57, *p *= 0.04, η_p_^2 ^= 0.10). Women (*M *= 16.55, *SE *= 2.03) omitted answers on significantly more PASAT items than men (*M *= 10.92, *SE *= 1.46).

### Differences between the ADHD subtype and NC groups

#### Wechsler WM measures

An analysis of the WM measures from the Wechsler scales identified a main effect of group on WM performance on the LNS test (*F*(2,45) = 4.32, *p *= 0.02, η_p_^2 ^= 0.13) (see Table 4 for means and SEs). Pairwise comparisons found that the ADHD-IA (*p *= 0.008) and the ADHD-CT group (*p *= 0.03) committed more errors than the NC group. A comparison between groups on the Digit Span test was not significant for a main effect (*F*(2,45) = 2.79, *p *= 0.07, η_p_^2 ^= 0.05) but showed the same pattern. Exploratory post hoc comparisons indicated that performance by the ADHD-IA group was worse than the NC group (*p *= 0.02), although there was no significant difference between the NC and ADHD-CT groups. There was no main effect for group on Arithmetic or Spatial Span tests. There were no significant group differences on the Wechsler tests between the two ADHD subtypes.

**Table 4:** Working memory performance for ADHD subtypes and normal controls

Measure							
							
	ADHD-CT (*n *= 17)		ADHD-IA (*n *= 16)		NC (*n *= 18)		
	
	*M*	*SE*	*M*	*SE*	*M*	*SE*	Post hoc comparisons with covariates
Arithmetic	11.65	0.58	11.75	0.60	13.39	0.56	ns
Digit Span	11.71	0.66	11.12	0.68	13.39	0.64	NC>ADHD-IA (*p *= .02)^b^
Letter-Number-Sequencing	11.65	0.69	11.31	0.71	14.33	0.67	NC>ADHD-CT, ADHD-IA (*p *= 0.03)
Spatial Span	11.59	1.02	11.00	0.42	11.94	0.53	ns
							
PASAT Averaged over ISI^a^	(*n *= 16)		(*n *= 14)		(*n *= 17)		
Correct	31.66	2.17	31.64	2.33	38.81	2.11	ns
Omission Errors	15.33	1.97	15.59	2.10	8.22	1.91	NC<ADHD-CT (*p *= 0.008)
Longest Sequence	8.25	2.58	12.31	2.62	16.91	2.50	NC<ADHD-CT (*p *= 0.02)

*Note*. NC = normal control, ADHD-IA = inattentive type, ADHD-CT = combined type.

^a^In some cases on the PASAT there were missing data due to technical problems in the computer recording responding. This included missing data for one ADHD-CT participant, two ADHD-IA participants, and one NC participant for PASAT measures only.

^b^Exploratory analyses

#### PASAT measures

On the Number Correct variable for the PASAT the differences between groups failed to show a significant effect (see Table 4 for PASAT data). On the Omission Error variable for the PASAT there was a main effect for Group (*F *(2,38) = 4.13, *p *= 0.02, η_p_^2 ^= 0.18). Post hoc pairwise comparisons suggested that the ADHD-CT group made more omission errors compared to the NC group (*p *= 0.008), however the difference in omission errors between the ADHD-IA and NC group was not statistically significant (*p *= 0.07). A significant main effect for Group emerged on the Longest Sequence variable (*F*(2,36) = 3.22, *p *= 0.05, η_p_^2 ^= 0.15). The post hoc pairwise comparisons indicated that the NC group generated significantly longer sequences of consecutive correct responses than the ADHD-CT group (*p *= 0.02), whereas there was no significant ADHD-CT versus ADHD-IA group difference.

### Group by gender interactions

The only group by gender interaction effect was on the PASAT Omission Error variable (*F *(1,38) = 3.24, *p *= 0.05, η_p_^2 ^= 0.15). There were no significant differences in omission errors between the groups in the female sample; the difference between groups in omission errors for the male sample approached significance (*F*(2,25) = 2.66, *p *= 0.09, η_p_^2 ^= 0.17). Exploratory analyses revealed that for the male group, pairwise comparisons showed that the ADHD-IA group generated significantly more omission errors than the ADHD-CT group (*p *= 0.02).

### Effect of ISI on PASAT performance

There were no main effects of ISI on any of the PASAT variables. However, there was a 3-way interaction of Group by ISI by Gender on the Omission Error variable (*F*(4.8,91.9) = 2.55, *p *= 0.03, η_p_^2 ^= 0.12). This 3-way interaction suggested the need to explore the effect of ISI presentation rate and group within each gender. The ANOVA for the male group only showed a significant ISI by group interaction (*F*(4.7,12.65) = 3.96, *p *= 0.004, η_p_^2 ^= 0.24). The overall male group was affected by the length of the ISIs in a linear fashion with shorter ISIs resulting in more errors (*F*(1,25) = 4.06, *p *= 0.05, η_p_^2 ^= 0.14). There was also a significant linear ISI by group interaction (*F*(2,25) = 8.67, *p *= 0.001, η_p_^2 ^= 0.41). The data indicate that as the presentation rate quickened for the PASAT, male subjects in the ADHD groups demonstrated a shallower slope in response to the faster ISIs than the NC group. Responding in the ADHD-IA group demonstrated the steepest slope, showing a greater number of omission errors at each ISI change (beginning at 2.4s to 2.0 s), whereas the ADHD-CT group did not show an effect of ISI until the ISI changed from 2.0 s to 1.6 s. There were no significant findings for the female group.

## Discussion

The bulk of the results from this study did not support the hypothesis that there would be WM differences between ADHD subtypes. There was partial support for the evidence of WM differences between the ADHD and NC groups and between ADHD subtypes in the male sample. The data revealed significant gender differences on WM performance for some measures and these gender differences may have obscured some of the WM impairments hypothesized to occur in the ADHD groups. The effect of these gender differences will be expanded upon below.

The findings from the LNS task were the strongest, suggesting that the processing load for individuals with either ADHD subtype may be more taxing when multiple executive functions are required such as in LNS which demands sequencing, manipulation of information, processing speed, auditory memory and perhaps visual-spatial imagery [51]. On Digit Span – a test related to LNS – there was some suggestion via exploratory analyses that the ADHD-IA group, but not the ADHD-CT group, performed more poorly than the NC group. These data suggest that there may not be an absolute difference in subtype performance, but instead a dimensional interpretation of WM deficits experienced by the ADHD-CT and ADHD-IA subtypes. The added processing requirements and need to keep more than one type of stimuli on-line during task manipulation for LNS may explain why both ADHD subtypes demonstrated significant differences from the NC group in this test relative to the less complex Digit Span subtest.

Neither the Arithmetic or Spatial Span subtests differentiated any of the groups. Arithmetic processing as assessed by the WAIS may be less sensitive to detecting WM deficits in ADHD because the task does not require as much juggling of potentially interfering stimuli inherent to the task as do the other WM tasks used. Similarly, performance on the Spatial Span test may not depend upon the same processing speed and verbal rehearsal strategies associated with other WM tests that revealed ADHD versus NC differences [52]. It is not clear if the lack of differences on Spatial Span are due to factors specifically related to the task or factors related to spatial versus nonspatial WM tasks. A meta-analysis on WM impairment in children and adolescents [15] suggests that children with ADHD are more impaired on spatial tasks rather than verbal. A meta-analysis in adults with ADHD [19], however, found that neuropsychological functioning in adults is more affected by ADHD on tasks involving a clear WM and verbal memory component than a task like Spatial Span. These data suggest the need for greater replication across a variety of WM tasks and comparisons between tasks measuring the extent of verbal component within the task, whether they are labeled "spatial" or "verbal", and comparisons across developmental levels.

On the PASAT we did not find the expected main effect for ISI. A recent review of the PASAT [26], however, supports our results, showing that faster ISIs across several studies did not impair performance or enhance sensitivity for between-group differences between control and clinical populations. The absence of a main effect of ISI may be undetectable in our sample because ISI effects appeared to depend on gender and group membership.

The Longest Sequence of Consecutive Correct Answers and Omission Error measures demonstrated ADHD versus NC differences on the PASAT. The ADHD-CT group performed most poorly of the groups on the Longest Sequence measure. This measure may reflect the dual dimension impairment espoused by DSM-IV for ADHD, Combined Type with performance suffering from impairment due to response inhibition combined with problems in inattention, on a score that is sensitive to both incorrect errors and omissions. Performance between the ADHD-CT and ADHD-IA groups on the Omission Error variable were differentially affected by gender and ISI presentation rate. With regard to gender, the combined male and female ADHD-CT group performed slightly worse than the ADHD-IA group, and was statistically significantly different from the NC group. By contrast, the same analysis among male participants only suggested that the ADHD-IA group performed worse than participants in the NC and ADHD-CT groups. We are speculating that the poorer performance in the ADHD-CT sample that included both men and women was substantially affected by errors produced by the female ADHD-CT participants. With regard to ISI, both male ADHD groups appeared to be affected by presentation rate, but the ADHD-IA group seemed most affected by shortening of the ISI. Faster presentation rate most likely increases processing load, and these data suggest that WM performance in ADHD-IA males may be more influenced by processing speed demands than the ADHD-CT group. These findings are consistent with earlier studies [5,25] suggesting that individuals with the ADHD-IA subtype may exhibit greater processing speed deficits. An understanding of the specific effect of gender on general omission errors and of the effect of presentation rate on omission errors according to ADHD subtype will require larger samples with an equivalent number of male and female participants. Research on stereotype threat, however, may shed some light specifically on the interaction effects between gender and subtypes for the PASAT variables. Stereotype threat research (e.g., [53] shows that women tend to underperform on mathematical tests if they think their performance is diagnostic of their ability. Furthermore, this underperformance may be due to a decrease in WM capacity in women if they are perceiving a stereotype threat [54]. It is possible that both the ADHD and NC women expected to do poorly on the PASAT because it involves mathematical operations and is also perceived as a frustrating, anxiety-provoking task by many [26]. Women also tended to perform more poorly than men on two other tasks involving numbers, LNS and DS, although neither actually involves arithmetic. These tasks are also not typically considered as stressful as the PASAT. Perhaps the combination of specific task characteristics of the PASAT precipitated a stereotype threat and interacted to produce different patterns of responding between gender and subtype. Future projects with the PASAT should specifically measure test anxiety associated with performance, exploring gender and subtype differences.

In some instances in this study, performance by NC females was equivalent to the ADHD females in both subtypes, and worse than the male participants of either subtype. The addition of female participants to the overall group appeared to obscure differences between the ADHD subtypes and NCs on some measures. The normal control literature on gender differences in WM may assist in interpreting our data, although larger samples of female participants are needed to confirm the existence of a gender effect on these measures. The examination of the relationship between gender and WM performance in the normal adult population appears to be understudied [55]. Some studies suggest there are no differences in spatial WM [56] between genders; however, others found men do perform better than women on visuo-spatial dependent memory tasks [55] if they require active manipulation but not passive storage [57]. Women do appear to perform more strongly than men on episodic memory tasks [55,58]. Research on gender differences in strategy use for arithmetic problems may shed some light on our findings. A study on young children [59] found girls were less capable than boys in using a retrieval mechanism to solve arithmetic problems from memory. Thus, WM tasks requiring a retrieval strategy, as many of the tasks did in our study, may be more sensitive to gender differences.

Processing speed load may also influence task performance. Speck et al. [41] found that women may respond more slowly on WM tasks. Many of the tasks used in this study were highly dependent on speed of processing, the PASAT in particular. One other published study [49] using the PASAT reported significantly higher performance in males. Another study found [48] that men outperformed women, but not significantly so. Still others [60-63] reported no gender differences on the PASAT. Thus, the poorer performance of women in all groups in this study may be related to the speed of processing load inherent in the tasks.

To date, research on gender differences in neuropsychological performance among individuals with ADHD is limited. Two studies [31,64] reported no gender differences among adult ADHD samples on several neuropsychological tests. In pediatric studies, the findings generally indicate both girls and boys with ADHD show executive functioning deficits compared to their control counterparts [35,36]. However, there is research indicating that ADHD girls do not perform significantly more poorly than controls on many tasks, suggesting there might be a gender difference between girls and boys [65]. Arnold [66] highlights the need for additional research in this area, suggesting that there may be differential symptom presentation that could affect performance across genders. Our data suggest the need for replication in larger healthy control and ADHD samples to explore the potential role of gender, performance expectations, and ADHD on WM functioning.

Overall, these results indicate that both subtypes demonstrate WM deficits on some tasks. While there were hints that the subtypes produced discrepancies on various measures of WM, these differences were not strong enough to indicate that the pattern is beyond dimension of impairment. The results from this study suggest it is important for future studies to tease apart the components used in WM tasks to better isolate how the specific processes could relate to the primary symptoms associated with the different subtypes. It is also important to recognize that both the ADHD-IA and ADHD-CT groups could demonstrate WM deficits, yet show those deficits for different reasons. At the level of the phenotype, the subtypes could be similar, yet at the neural level they could be quite different. For example, WM deficits are common not just to ADHD but to many psychiatric illnesses, including schizophrenia [67,68]. The presence of WM deficits in different clinical populations suggests there is a need for a better understanding of the underlying cognitive processes and neural underpinnings presenting as impairments in WM task performance. An imaging study might identify how the pattern of WM impairments associated with each subtype might or might not be linked to different neuroanatomy. Imaging data would lend more support to theories hypothesizing that the ADHD subtypes are better conceptualized as distinct disorders rather than variants of the same disorder or degree of impairment within particular brain regions.

### Limitations and future research

The study sample was small with high IQs, which suggests the need for further studies with larger samples and ranges of intellectual functioning. Additional studies also would benefit from including a clinical control group to provide a more critical test of how sensitive the various measures are to ADHD deficits specifically. Studies using WM paradigms with incremental difficulty loads on manipulation and processing speed requirements and equivalently large samples of females and males may be able to tease apart the effects on these variables of ADHD subtypes and gender. The small sample size and even smaller number of participants by gender within each group limited the conclusions that could be drawn in relation to gender and subtype.

Future studies with an emphasis on refining the diagnostic criteria for the subtypes of ADHD will also contribute to the understanding of ADHD. The question of symptom threshold and 'purity' of subtypes is a critical one. The recent research on the Sluggish Cognitive Tempo (SCT) type of ADHD-IA individual [69] may be helpful in designing future studies that examine subtype differences. Such individuals may constitute a more pure form of the ADHD-IA subtype that is categorically different from the ADHD-CT type [69]. Therefore, studies using an SCT criterion may be more sensitive to subtype differences.

## Conclusion

WM deficits are particularly evident in tasks comparing normal control adults to adults with ADHD when tasks requiring significant processing speed are used. Differentiation in WM performance between adults with the Inattentive versus Combined Type of ADHD are less pronounced and may be obscured when groups of male and female subjects are combined. Males with the ADHD-IA subtype may experience greater WM deficits with higher processing speed loads. These data suggest future studies should explore the role of gender on WM functioning in both ADHD and normal control populations.

## Declaration of competing interests

The author(s) declare that they have no competing interests.

## Authors' contributions

RBH and JBS conceived of the study. All authors contributed to writing the manuscript. RBH collected the testing data; JBS collected a subset of the interviews and reviewed all of the interview and rating scale data. RBH performed the preliminary data analysis and JBS and DRM performed the final analyses. All authors read, provided feedback and approved the manuscript.
